# Suppression subtractive hybridization analysis reveals expression of conserved and novel genes in male accessory glands of the ant *Leptothorax gredleri*

**DOI:** 10.1186/1471-2148-10-273

**Published:** 2010-09-08

**Authors:** Angelika Oppelt, Fernanda C Humann, Marion Fuessl, Sergio V Azevedo, David S Marco Antonio, Jürgen Heinze, Klaus Hartfelder

**Affiliations:** 1 Biologie I, Universität Regensburg, Universitätsstr. 31, D-93040 Regensburg, Germany; 2 Departamento de Biologia Celular e Molecular e Bioagentes Patogênicos, Faculdade de Medicina de Ribeirão Preto, Universidade de São Paulo, Av. Bandeirantes 3900, 14049-900, Ribeirão Preto, Brazil; 3 Departamento de Genética, Faculdade de Medicina de Ribeirão Preto, Universidade de São Paulo, Av. Bandeirantes 3900, 14049-900, Ribeirão Preto, Brazil

## Abstract

**Background:**

During mating, insect males eject accessory gland proteins (Acps) into the female genital tract. These substances are known to affect female post-mating behavior and physiology. In addition, they may harm the female, e.g., in reducing its lifespan. This is interpreted as a consequence of sexual antagonistic co-evolution. Whereas sexual conflict abounds in non-social species, the peculiar life history of social insects (ants, bees, wasps) with lifelong pair-bonding and no re-mating aligns the reproductive interests of the sexes. Harming the female during mating would negatively affect male fitness and sexual antagonism is therefore not expected. Indeed, mating appears to increase female longevity in at least one ant species. Acps are presumed to play a role in this phenomenon, but the underlying mechanisms are unknown. In this study, we investigated genes, which are preferentially expressed in male accessory glands of the ant *Leptothorax gredleri*, to determine which proteins might be transferred in the seminal fluid.

**Results:**

By a suppression subtractive hybridization protocol we obtained 20 unique sequences (USs). Twelve had mutual best matches with genes predicted for *Apis mellifera *and *Nasonia vitripennis*. Functional information (Gene Ontology) was available only for seven of these, including intracellular signaling, energy-dependent transport and metabolic enzyme activities. The remaining eight USs did not match sequences from other species. Six genes were further analyzed by quantitative RT-PCR in three life cycle stages of male ants. A gene with carboxy-lyase activity and one of unpredicted function were significantly overexpressed in accessory glands of sexually mature males.

**Conclusions:**

Our study is the first one to investigate differential gene expression in ants in a context related to mating. Our findings indicate that male accessory glands of *L. gredleri *express a series of genes that are unique to this species, possibly representing novel genes, in addition to conserved ones for which functions can be predicted. Identifying differentially expressed genes might help to better understand molecular mechanisms involved in reproductive processes in eusocial Hymenoptera. While the novel genes could account for rapidly evolving ones driven by intra-sexual conflict between males, conserved genes imply that rather beneficial traits might get fixed by a process described as inter-sexual cooperation between males and females.

## Background

Substances produced by the male accessory glands (MAGs) of insects and transferred into the female genital tract during mating are known to reduce pathogen transmission, to form mating plugs or spermatophores, and to be important in sperm competition. In addition, they trigger fundamental changes in female physiology, behavior, and reproduction [1-4]. The major biologically active components of MAG secretions are carbohydrates, lipids, and in particular accessory gland proteins (Acps) [1].

In *Drosophila melanogaster*, more than 100 Acps have been identified [5]. They play roles in the regulation of female receptivity [6-9], defense against bacteria [10], the activation of the female immune system [11,12], the efficiency of sperm storage [13,14], and the stimulation of ovulation and egg maturation [6,8,9]. They are also decisive for the fertilization of eggs, as shown in experiments in which the activity of sperm of infertile mutant males lacking MAGs could be restored by subsequently re-mating females with males that provided Acps but no sperm [15].

In addition to stimulating immediate female fertility, MAG products appear to negatively affect female lifespan [16,17]. This reflects inter-sexual conflict [18]: the reproductive interests of males and females concerning the consequences of a mating act may differ, in particular when females later re-mate with other partners. Males prefer the female to maximize short-term investment into the offspring resulting from the present copulation, while females may benefit more from saving resources for later reproduction with other males. Sexual conflict may result in sexually antagonistic co-evolution and Acps indeed appear to evolve very rapidly, as predicted from the ensuing arms race [5,19-23].

Compared to the wealth of information on accessory gland products of drosophilids, studies on other insect species are seriously lagging behind [1]. This is particularly the case for eusocial Hymenoptera, despite the special implications that their life style and evolutionary history have on sexual conflict. In contrast to *Drosophila *and other non-social insects, eusocial Hymenoptera are characterized by life-long pair-bonding, even though the males frequently die after mating [24-26]. Investigations have shown that monogamy is the ancestral state of all truly eusocial organisms [27], and lifetime monogamy seems to be a universal precondition for the evolution of obligate eusociality [24,25]. The queens of ants, eusocial bees, and wasps indeed mate only once or a few times early in their adult lives without ever re-mating again. Sperm received during these matings is stored for months, years, or even decades, and utilized to fertilize eggs throughout the queens' lives. Furthermore, queens first rear large numbers of sterile workers from fertilized eggs before switching to the production of female sexuals. Hence, in contrast to males of non-social insects, eusocial insect males are expected to avoid harming the female, as this would reduce the total amount of female sexuals produced and, consequently, male reproductive success. Instead, males should have evolved mechanisms that promote female performance. Even where multiple mating has evolved after the monogamy window has been passed, competition among ejaculates [28] is not expected to negatively affect female life span and future fecundity. Inter-sexual conflict might thus be replaced by "inter-sexual cooperation", though intra-sexual conflict might still evolve. Indeed, ant males appear to positively affect queen life span: regardless of being mated with a fertile or sterilized male, mated queens of *Cardiocondyla obscurior *lived considerably longer than virgin queens [29].

Like in other insects, MAG products of eusocial Hymenoptera are involved in the formation of mating plugs [30-32] and ejaculate (or eventually sperm) competition [28,33]. In addition, they presumably elicit rapid changes in the physiology and behavior of freshly mated queens, e.g., the shedding of wings, reluctance of mating again, and changes in cuticular hydrocarbons [34]. It is likely that MAG products, and in particular Acps, are also involved in the life-prolonging effects of mating [29]. Nevertheless, very little is known about Acps in eusocial Hymenoptera other than the honey bee, *Apis mellifera *[35-38]. We therefore examined, which genes are differentially expressed in the male accessory glands of the ant *Leptothorax gredleri*.

Virgin queens of *L. gredleri *attract males through "female calling", i.e., they climb up grass stems or similar, elevated positions near their maternal nests and release droplets of a sex pheromone from their poison glands [39-41]. During mating, sperm is transferred within a gelatinous substance, functionally similar to a spermatophore. The latter stays inside the female's genital tract for several hours, while the sperm is slowly transferred into the spermatheca [42]. Females have been observed to copulate with multiple males but it is unclear whether ejaculate is transferred during all these matings. Genetic analyses document single paternity of their offspring [40].

In order to obtain information on gene products specifically produced by the accessory glands of *L. gredleri *males we generated a suppression subtractive library for transcripts using a Representational Difference Analysis (RDA) protocol. Such an approach enriches tissue-specific transcripts (here: MAGs) by removing from the library sequences that are shared with control tissue (here: the body of the males after excision of MAGs).

## Results

### RDA library characteristics and bioinformatic analyses

To detect differentially expressed genes in *L. gredleri *MAGs, we sequenced 192 clones from the subtracted gland library, resulting in 288 sequencing reads, of which 158 reads representing 130 different clones were of appropriate quality (Phred quality ≥ 20). After quality analysis and following CAP3 assembly we obtained 12 contigs and 8 singlets as Unique Sequences (USs). The sequences were submitted to GenBank (accession numbers GT897811 until GT897966).

BLASTx analyses of the USs (Table 1) revealed significant matches (E-scores < e^-5^, a cut-off level frequently used in EST projects; [43]) for 12 USs. The remaining 8 USs showed no significant matches to genes of other species.

**Table 1:** EST sequences from RDA libraries of accessory glands cDNA from *Leptothorax gredleri*

Gene (predicted)	ESTs #	Base pairs	Organism	e-value	Gene Ontology attributes
*leukocyte-antigen-related-like*	2	370	*Apis mellifera*	4e^-37^	Protein tyrosine phosphatase activity, axon guidance, cell adhesion, motor axon guidance, nervous system development, oogenesis, photoreceptor cell morphogenesis, protein amino acid dephosphorylation, R7 cell development, regulation of cell shape, retinal ganglion cell axon guidance
*kokopelli*	2	263	*Apis mellifera*	1e^-45^	Cyclin-dependent protein kinase regulator activity
*ATP synthase gamma chain, mitochondrial precursor*	5	170	*Apis mellifera*	9e^-09^	Hydrogen-exporting ATPase activity, phosphorylative mechanism; hydrogen ion transporting ATP synthase activity, rotational mechanism; proton-transporting ATPase activity, rotational mechanism
*nervana 2*	7	215	*Apis mellifera*	4e^-19^	Cation transmembrane transporter activity, sodium:potassium-exchanging ATPase activity
*CG1486*	40	249	*Apis mellifera*	4e^-17^	Carboxy-lyase activity, pyridoxal phosphate binding
*ribosomal protein LP1*	1	167	*Apis mellifera*	6e^-78^	Structural constituent of ribosome
*CG6910*	1	197	*Apis mellifera*	2e^-08^	Inositol oxygenase activity, iron ion binding
*SJCHGC05576*	3	165	*Nasonia vitripennis*	1e^-05^	
*LOC100123166*	1	101	*Nasonia vitripennis*	4e^-09^	
*ciao-1*	1	184	*Apis mellifera*	7e^-17^	
*CG32432*	1	212	*Apis mellifera*	1e^-05^	
*heat shock factor binding protein 1-like*	12	159	*Apis mellifera*	3e^-18^	
					
US1	3	158	No match		
US2	5	146	No match		
US3	10	173	No match		
US4	28	179	No match		
US5	5	183	No match		
US6	1	167	No match		
US7	1	180	No match		
US8	1	97	No match		

Shown are the predicted gene, the number of ESTs assembled to each US, US size in bp, the respective organism, followed by the respective similarity index (e-value) and corresponding Gene Ontology terms obtained from Flybase http://flybase.bio.indiana.edu/.

Most of the ESTs (40) clustered to a sequence corresponding to an *Apis mellifera *predicted gene similar to CG1486-PA. Based on domain predictions, the fly ortholog of this gene is annotated in Flybase as being involved in carboxylic acid metabolism. Another US represented by 12 ESTs showed similarity to a heat shock factor binding protein 1-like from *Apis mellifera*, with the corresponding CG5446-PA in *Drosophila melanogaster*. Further USs were also formed by clustering considerable numbers of ESTs (Table 1), such as US4 consisting of 28 ESTs, and US3 composed of 10 ESTs. None of these transcripts, however, presented matches when submitted to BLASTx analysis.

In functional terms, the searches for orthologs revealed that, seven of the *L. gredleri *USs corresponded to functionally defined genes (Table 1), with best hits to predicted *A. mellifera *proteins. By judging from Gene Ontology attributes, two are expected to be involved in energy-dependent transport processes, such as the ATP synthase gamma chain and Nervana 2, a sodium/potassium-transporting ATPase subunit. The Leukocyte-antigen-related-like protein may function as a protein tyrosine phosphatase, while the *kokopelli *gene has a cyclin-dependent protein kinase regulator activity and is involved in male germ-line stem cell division. The final two sequences with predicted molecular functions are CG6910-PA, with inositol oxygenase activity, and an *Apis mellifera *ribosomal protein LP1 (RpLP1) given as being a structural constituent of ribosomes.

Still unknown are biological processes or molecular functions for the five remaining sequences with BLASTx matches. SJCHGC05576 and LOC100123166 are both predicted proteins of the parasitic wasp *Nasonia vitripennis*, and Ciao-1 and CG32432-PA and heat shock factor binding protein 1-like are predicted proteins for *A. mellifera*. Neither of these had matches with *Drosophila *genes.

### Quantitative RT-PCR analysis of RDA library genes

The RDA method is applicable to systems where genomic information is either completely lacking or still in an incipient stage. RDAs are able to detect both known and so far unknown transcripts, including regulatory RNAs. However, being a high throughput approach, transcripts indicated by RDA as differentially expressed should be further analyzed by specific gene-expression analyses, such as quantitative RT-PCR (qRT-PCR). Thus, we designed gene-specific primers for 19 of the 20 RDA products. One product contained repeats, and so it was not considered suitable for primer design. For six of the 19 products we obtained primers, shown in Table 2, that reliably amplified specific products and, thus, were suitable for qRT-PCR. These were primers for the genes *leukocyte-antigen-related-like *(*leukocyte*) and *CG1486*, representing two genes with BLASTx matches and predicted functions, for *ciao-1 *and *CG32432*, being genes with matches but of unknown function, and the final two being for US3 and US7, representing no-match sequences.

**Table 2:** qRT-PCR primers sequences with their target genes (when known)

Target genes for qRT-PCR		
*leukocyte-antigen-related-like*	LgCon1F LgCon1R	5' TTCGTGCGCGTTCTGCTTACC 3' 5' GCATCATCGCAGAATACCGGTC 3'
*CG1486*	LgCon12F LgCon12R	5' GAAGCGGAAGATGTGGC 3' 5' GCCAGCTCTAACCGAAAC 3'
*ciao-1*	S3BO6-F S3BO6-R	5' CCTAATGATATCGTCACCGC 3' 5' GGAATATAAACCTGGGAACG 3'
*CG32432*	S8H10-F S8H10-R	5' GATCGATTCGAGCAGAGACA 3' 5' TGTTTATCGGGAATCGTTTC 3'
US3 - no match	LgCon9F LgCon9R	5'CTCATCCCTGGGACTTGCAC 3' 5'GTGGACCCCGAGAAATAGA 3'
US7 - no match	S6FO5-F S6FO5-R	5' GTTACGCTTTACGCAACGAG 3' 5' CTTTCATTCCACGGCTCATC 3'
elf-1 alpha	LG-ELF1aF LG-ELF1aR	5' CATGATCACCGGTACCTCG 3' 5' CCAGCATGTTGTCTCCGTG 3'

**Figure 1:**
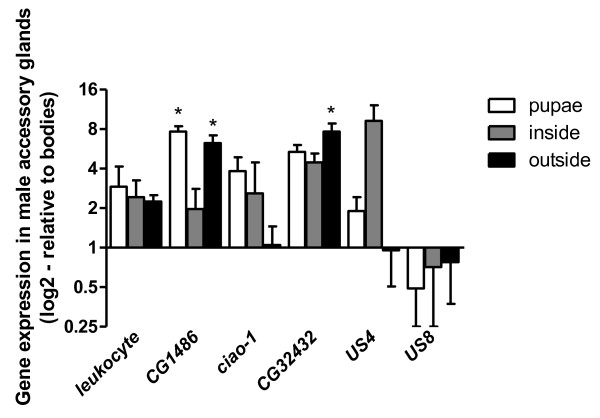
**Gene expression in male accessory glands**. Relative expression of *leukocyte-antigen-related-like, CG1486, ciao-1, CG32432*, US3 and US7 transcripts in male accessory glands of the ant *Leptothorax gredleri *relative to body carcasses. The y-axis, drawn through 1, corresponds to the expression level in the body carcass (control tissue) of the respective life cycle stage, these being late male pupae, males inside nests and males outside nests. Shown are fold change expression ± SEM for five biological replicates. Statistically significant differences (REST randomization significance test, *P *< 0.05) are indicated by asterisks.

For five of the six genes we could show that their mean transcript levels are at least two-times higher in the *L. gredleri *MAGs relative to body carcasses, this holding for all three of the analyzed male life cycle stages, except for ciao-1 in the males running outside the nest that were sexually mature (males outside nests) samples (Figure 1). For the sixth gene, US7, overexpression was not validated. This may, thus, represent false positive RDA identification, represented by a single sequencing read (Table 1).

When testing for statistical significance of differential expression by randomization tests built into REST software we could show significant overexpression in two genes. *CG1486*, a gene with predicted carboxy-lyase activity, was not only represented by the highest number of ESTs (Table 1), but was also confirmed as significantly overexpressed in MAGs of dark-pigmented male pupae (male pupae; *P *= 0.010) and sexually mature males (males outside nests; *P *= 0.015). The other gene, *CG32432*, was shown to be significantly overexpressed in sexually mature males (*P *= 0.037) (Figure 1). For all of these five genes biological variation in expression levels was considerable, a problem frequently encountered in qRT-PCR analyses, even with highly controlled cell cultures [44].

## Discussion and conclusions

Using the RDA method we successfully identified a set of 20 genes as being differentially expressed in MAGs of the ant *L. gredleri*. Unfortunately, a fully sequenced ant genome is not yet available, so an annotation against a genome was not feasible. Such an annotation would be needed to identify proteins that are secreted into the lumen of *L. gredleri *MAGs, as their RNAs should have hydrophobic amino-terminal signal sequences [45]. For example, Swanson et al. [22] found that 24% of the 212 identified genes in their study of *Drosophila *male reproductive proteins have such a putative signal sequence.

All best hit matches were against *A. mellifera *or *N. vitripennis *predicted genes, emphasizing the importance of these hymenopteran reference genomes in future gene expression studies in eusocial insects. Nevertheless, most of our ESTs represented functionally undefined genes or ones without matches to known genes. At first sight, this might indicate rapid evolution and species specificity in MAG secretion products, similar to what has been shown for MAGs of *Drosophila *[22], where about 50% of the *Drosophila *Acps have no homologs outside the drosophilid species complex. When comparing the genes predicted to be differentially expressed in MAGs of *L. gredleri *with those identified in a proteome analysis of honey bee seminal fluid [38] we did not find any matching gene products for the set with GeneOntology functional attributes. This finding would also be in favor of a rapid evolution hypothesis for MAG products. Queens of *Leptothorax *are predominantly singly-mated [40,46], but occasional multiple mating might introduce intra-sexual conflict and thus increase the evolutionary tempo of seminal fluid proteins. In eusocial species with multiple mating, the beneficial effect of seminal fluid on sperm survival is reduced when seminal fluid is mixed with alien sperm [28]. However, this may actually serve the interests of the queen rather than inflicting damage. Furthermore, the queen appears to be in control of ejaculate competition, since the negative impact of seminal fluid on foreign sperm can be neutralized by secretions from the queen sperm-storage organ [28]. In this case, intra-sexual conflict seems to be controlled by inter-sexual cooperation.

Concerning our studies, nothing is as yet known on the molecular nature of MAG secretions of other ant species, and with *Apis mellifera *and *Nasonia vitripennis *being the closest species for genomic comparisons, the percentage of functionally undefined genes and of no-match ESTs might simply reflect the large phylogenetic distance separating ants, bees, and parasitic wasps. Speculating about the speed of the evolution of *L. gredleri *Acps would, at this point, be premature, especially since, 2D-electrophoreses of Acps suggest that they are conserved in *L. gredleri *and related taxa relative to, e.g., muscle proteins (M. Fuessl, A. Oppelt, J. Heinze, unpublished).

Furthermore, not all seminal fluid genes can be subject to rapid evolution, because successful reproduction must be assured. In a cross-species comparison of *Drosophila *MAG protein encoding genes, Mueller et al. [23] found not only rapidly evolving but also conserved genes, and functionally similar proteins may be represented and relevant in the seminal fluid of other animals [4]. Members of protein classes conserved across organisms in seminal fluids may reflect their general importance in reproductive physiology [4] and might therefore be beneficial for both males and females. In this context, immune defense proteins are of special interest. MAGs secrete proteins of antimicrobial activity [10] and proteins, which regulate the expression of genes with antimicrobial activity in females after sperm transfer [11,12,47]. Such Acps might successively protect sperm from microbial attack in the reproductive tracts of both sexes, as well as coat eggs when they are laid. A *L. gredleri *EST, whose ortholog *CG6910 *is known to be upregulated in *D. melanogaster *females after mating [47], might play such a role. The *Drosophila *gene is annotated as possibly showing oxidoreductase activity and being downregulated after bacterial infection. For the elucidation of its function in ants, it should be of interest to see whether the gene similar to *CG6910 *is also differentially expressed in virgin and mated *L. gredleri *queens. Females are not 'silent partners' when it comes to seminal fluids and it is always an interplay of male and female factors that coordinates the gametes for fertilization [1,28,48].

The CG32432 protein predicted in the honey bee genome as a *Drosophila *ortholog with undefined function was found to be significantly overexpressed in sexually mature *L. gredleri *males, and one gene similar to CG1486-PA was found to be overexpressed in two male life cycle stages. This latter protein is predicted to have a carboxy-lyase activity and might be involved in carboxylic acid metabolism, catalyzing the non-hydrolytic addition or removal of a carboxyl group to or from a compound. Carboxylic acids are widespread in nature, giving rise to fatty acids. Fatty acids, such as palmitic-, linoleic-, oleic-, and stearic-acids, have been described as components of the mating plug in the bumblebee *Bombus terrestris*, which form a physical barrier [30] and prevent re-mating [31]. Mating plugs were also found in fire ants [32], queenless *Dinoponera *ants [49], attine ants [50], stingless bees [51], and in the honey bee. In the latter, however, they do not play a function as a plug, but rather as a mating sign that may even promote multiple matings [52]. In *L. gredleri*, the existence of a spermatophore that could be comparable to a mating plug was already shown [42]. The overexpression of a gene with carboxy-lyase activity in the late pupal stage, when the accessory glands are already formed, and in sexually mature males found outside the nest supports the idea that accessory gland products are crucial for spermatophore formation in this species.

Taken together, our current study represents a first step addressing at a molecular level the reproductive interplay between *L. gredleri *males and their mates. MAG products identified in this study may play different roles, either as conserved or as species-specific proteins.

Understanding the molecular underpinnings of differences in reproductive strategies among non-social and eusocial species, and in the latter among monogamous (the majority of ants bees and wasps) and polygamous ones (e.g. the honey bees) will still require sequencing of MAG products in a range of species. Nevertheless, we believe that at this interface of "molecular communication" between the sexes, much can be gained towards comprehending the evolution of eusociality at the passage through the monogamy window [25,28], with inter-sexual conflict being replaced by inter-sexual cooperation.

## Methods

### Colony collection and male dissection

Colonies of *Leptothorax gredleri *Mayr 1855 (Hymenoptera: Formicidae) were collected from nest-sites at the edge of an abandoned army drill ground in Erlangen, Germany (49° 35' 09" N, 11° 02' 02" E). Each single colony was transferred into a three-chamber plastic box with a cavity between two microscope slides serving as a nest. Colonies were kept under standard rearing conditions [53,54] until they produced sexuals.

Males were dissected in chilled Beadle solution (128.3 mM NaCl, 4.7 mM KCl, and 2.3 mM CaCl_2_; [55]). The accessory glands were separated and the seminal ducts were removed. The intestine was removed to avoid contamination of libraries with microorganismal RNA and then the rest of the body served as reference tissue. Glands and the remaining body carcass were stored separately in RNAlater^® ^(Ambion) for subsequent RNA extraction. For library preparations, 40 pairs of glands were pooled for a single sample, to be subtracted against the carcasses of 20 bodies.

For qRT-PCR analyses we dissected male pupae ready to eclose, when they are already dark pigmented (male pupae), newly eclosed males collected from within the nest (males inside nests), and males running outside the nest that were sexually mature (males outside nests). Each sample consisted of at least 20 pairs of glands, to be compared against corresponding body carcasses. As the real time RT-PCR analyses were carried out as quintuplicates, this represented biological material of more than 100 males per life cycle stage.

### Representational difference analysis

RNA of both the glands and the remaining body from males (collected from within nests) was extracted with TRIzol (Invitrogen) according to the manufacturer's standard protocol. We used 2 μg of each RNA sample to generate the cDNA library. Reverse transcription and long distance PCR were carried out by employing the SMART PCR cDNA synthesis kit (Clontech), resulting in double-stranded cDNA.

For suppression subtractive hybridization we employed a cDNA RDA [56] adapted for applications in insects [57]. This strategy removes transcripts shared by the driver and tester populations and enriches the tester library for differentially expressed genes. An accessory gland library was generated using cDNA of accessory glands as the tester population. The latter was hybridized against cDNA of the remaining body carcass as the driver population. Double-stranded cDNAs (1 μg) were restriction digested with *Mbo*I (New England BioLabs), ligated to R-adapters [57], and PCR amplified following the protocol of Hubank and Schatz [58] to generate the respective cDNA representations. Enrichment of differentially expressed transcripts was achieved in two successive rounds of PCR amplifications that employed different adapters (J and N) and sequential subtractive hybridizations of the tester to an excess of driver cDNA, first in a **1:**100 and then in a **1:**800 ratio [58]. After each of these successive steps, cDNAs were purified (Illustra GFX PCR purification kit, GE).

cDNA from the accessory gland library after the second round of selection was used for ligation into pGEM^®^-T Easy Vector System (Promega) and transformation of *E. coli *DH5α chemocompetent cells. The cells were reared on solid LB containing ampicillin, X-Gal and IPTG. Selected clones were grown in liquid LB medium containing ampicillin. For the sequencing, the cells were lysed and the plasmids extracted and sequenced using Big Dye Terminator Cycle Sequencing Ready Reaction (Applied Biosystems) with M13 primers on an ABI-PRISM 3100 (Applied Biosystems) automated gene analyzer.

### Bioinformatic analysis

By means of the E-Gene annotation pipeline [59], sequencing reads were first filtered to detect and remove ribosomal RNAs and mitochondrial DNA. Subsequently, vector sequences were trimmed using Crossmatch. Read quality was checked and reads were assembled by the Phred-Phrap program module. Reads that had passed the quality check were next submitted to CAP3 assembly [60] to obtain USs. All contigs, and singlets were dynamically translated and compared by BLASTx to a non-redundant (nr) database (GenBank). ESTs with similarities to genes of known function in other organisms were clustered using Gene Ontology (GO) terms [61] attributed to their respective *Drosophila *orthologs. GO attributes were retrieved from the Flybase server http://flybase.org.

### Differential gene expression analysis by quantitative RT-PCR

For real time qRT-PCR analysis, gene-specific primers were designed for a set of selected USs using Gene Runner version 3.05 and Primer3 softwares.

As reference gene we chose elongation factor 1-alpha (*elf1-α*), a recommended control gene for honey bee qRT-PCR studies [62]. To obtain an *L. gredleri *sequence for *elf1-α *we aligned the corresponding sequences of *Leptothorax muscorum *(a direct sister species to *L. gredleri*) and *Temnothorax rugatulus *(a more distantly related ant species) (GenBank accession numbers ABK54792.1 and ABK54711.1, respectively) and designed primers to a conserved region. An *L. gredleri elf1-α *fragment was cloned and sequenced, and qRT-PCR suitable primers were designed (Table 2). For the qRT-PCR analyses, we used male pupae, males inside nests and males outside nests. Each sample consisted of a least 20 male bodies or glands. RNA of the glands and remaining bodies carcasses was extracted using TRIzol. The RNA samples were treated with 0.1 U DNaseI (Invitrogen) for 40 min to eliminate possible DNA contamination. First strand cDNA was produced using a SuperScript II (Invitrogen) protocol at 42°C for 50 min and 70°C for 15 min.

Quality and annealing temperatures of the gene-specific primers were tested in a temperature-gradient PCR protocol run in a PTC200 thermal cycler (MJ Research). Product length varied from 305 bp for *elf1-α *to 98 bp for the shortest of the *L. gredleri *singlets.

Subsequently, a dilution series was analyzed for each primer pair using a composite cDNA sample from bodies and glands of all stages in order to obtain standard curves for determining primer efficiencies. These and subsequent qRT-PCR amplifications were performed by using a SYBR Green (Applied Biosystems) protocol in an ABI Prism 7500 system (Applied Biosystems). The amplification protocol was 50°C for 2 min, 95°C for 10 min, 40 cycles of 95°C for 15 s and 60°C for 1 min. Subsequently, dissociation curves were acquired to check melting peak quality. For each life-cycle time point we ran five biological replicates, each assayed in technical triplicates, both for accessory glands and body carcasses.

Threshold cycle (Ct) values for each technical replicate were used to calculate 2^-ΔΔCt ^values [63] using the means of the five body carcass replicates of each life cycle stage for normalization of the respective gland samples. For statistical analysis, Ct values were used as input to randomization tests implemented in Relative Expression Software Tool (REST - [64]). *P *values ≤ 0.05 were considered as statistically significant.

## Authors' contributions

JH and KH conceived the study. KH, JH and AO designed the study. AO and MF collected and reared the males, dissected them and extracted the RNA. FCH and AO conducted the RDA, cloned and sequenced the fragments and conducted the bioinformatics analysis. SVA and AO designed the primers, MF, AO, FCH, SVA and DSMA tested the primers and conducted qRT-PCR. FCH analyzed the qRT-PCR results. AO, FCH, KH, and JH wrote the manuscript. AO and FCH contributed equally to the study. All authors read and approved the final manuscript.
